# Full Endoscopic Posterolateral Transarticular Lumbar Interbody Fusion Using Transparent Plastic Working Tubes: Technical Note and Preliminary Clinical Results

**DOI:** 10.3389/fsurg.2022.884794

**Published:** 2022-06-13

**Authors:** Yu Du, Fuling Jiang, Haiyan Zheng, Xudong Yao, Zhengjian Yan, Yang Liu, Liyuan Wang, Xintai Zhang, Liang Chen

**Affiliations:** ^1^ Department of Orthopedic Surgery, The Second Affiliated Hospital of Chongqing Medical University, Chongqing, China; ^2^ Department of Spine Surgery, Center of Orthopedics, Daping Hospital, Army Medical University (Third Military Medical University), Chongqing, China; ^3^ School of Nursing, Chongqing Medical and Pharmaceutical College, Chongqing, China; ^4^ Department of Orthopedic Surgery, Nan’an District People’s Hospital, Chongqing, China; ^5^ Department of Bone and Soft Tissue Oncology, Chongqing University Cancer Hospital, Chongqing, China

**Keywords:** FE-PTLIF, TLIF, conventional interbody cage, transparent plastic working tube, complication, learning curve

## Abstract

**Background:**

A series of full-endoscopic lumbar interbody fusions have been reported, but special fusion cages or operating instruments are often needed, and there are many complications in the operation and the learning curve is long. We have used a single portal endoscopic system for lumbar interbody fusion in a novel posterolateral transarticular approach, which will take advantage of the incision for pedicle screw insertion and avoid nerve root damage by using a transparent plastic working tube. The purpose of this study was to present the surgical technique of full endoscopic posterolateral transarticular lumbar interbody fusion (FE-PTLIF) and to analyze the preliminary clinical results.

**Methods:**

A total of 39 patients (17 men and 22 women; mean age [*x̅* ± *s*] 55.2 ± 12.2 years) have been enrolled in this retrospective study between March 2019 and January 2021 in the Second Affiliated Hospital of Chongqing Medical University. All patients were treated with full endoscopic lumbar interbody fusion via posterolateral transarticular approach with a transparent plastic working tube. Demographic characteristics, diagnosis, operative time, and estimated blood loss were evaluated. Intraoperative photo and perioperative imaging were recorded. The preoperative and postoperative clinical data were collected for statistical analysis.

**Results:**

The preliminary clinical follow-up data achieved good results. No patients had serious postoperative complications and none of these patients required revision surgery during the perioperative or follow-up period. We compared the visual analogue scale and Oswestry disability index scores before and after surgery. The differences were statistically significant (*P* < 0.05). The mean total blood loss (including drainage blood) was 54.4 ± 20.3 ml. The mean operative time was 130.5 ± 23.8 min. At the last follow-up, the fusion rate of the lumbar intervertebral space was 100%.

**Conclusions:**

This novel posterolateral transarticular approach and transparent plastic working tube can reduce the difficulty of the operation, so that the conventional intervertebral fusion cage [bullet-shaped polyetheretherketone (PEEK) nonexpandable fusion cage] and surgical instruments can be used in the full endoscopic lumbar intervertebral fusion surgery, which can reduce the cost and improve the efficiency of the operation.

## Introduction

Lumbar spinal fusion surgery has been well demonstrated to relieve pain and improve function and quality of life for many patients who suffered from lumbar degenerative disease (1, 2). There are a lot of methods in lumbar fusion surgery, including posterolateral lumbar fusion, posterior lumbar interbody fusion, transforaminal lumbar interbody fusion, direct lateral lumbar interbody fusion, anterior lumbar interbody fusion (ALIF), oblique lateral lumbar interbody fusion (OLIF), minimally invasive TLIF (MIS-TLIF), endoscopic approach for the lumbar interbody fusion, and full endoscopic lumbar interbody fusion (FELIF) (3–5). As the quality of life has become the main goal of health care, there is an increasing and critical demand for the development of minimally invasive spine surgery (MISS) techniques for lumbar fusion surgery. MISS has many advantages including lower risk of complications, lower risk of muscle damage, less pain, and faster recovery time (6, 7). Recently, among all MISS approaches, FELIF surgery has received substantial attention (8, 9).

We have used a single portal endoscopic system for lumbar interbody fusion in a novel posterolateral transarticular approach, which will take advantage of the incision for pedicle screw insertion and avoid nerve root damage by using a transparent plastic working tube. The purpose of this study was to present the surgical technique of full endoscopic posterolateral transarticular lumbar interbody fusion (FE-PTLIF) and to analyze the preliminary clinical results.

## Materials and Methods

### Preoperative Preparations

The chief Surgeons have started single portal percutaneous endoscopic spine surgeries in 2010, and all contributing authors have extensive experience in such percutaneous endoscopic surgeries as discectomy for lumbar disc herniation and decompression for lumbar stenosis by a transforaminal or an interlaminar approach. Before the clinical application of FE-PTLIF, we prospectively practiced such a surgery technique at 12 lumbar levels in four cadavers since 2018.

### Indication of FE-PTLIF

We initially only performed single-level fusion surgery from L3–4 to L5–S1. Indications of FE-PTLIF were the same as those for TLIF, including (1) lumbar disc herniation with segmental instability; (2) lumbar spinal stenosis with segmental instability; and (3) lumbar spondylolisthesis (less than Meyerding grade II). We did not perform endoscopic fusion in cases of infection, spondylodiscitis, vertebral fractures, severe central canal stenosis, or spondylolisthesis greater than grade III.

### Surgical Technique

#### Position, Anesthesia, Approach, and Percutaneous Screw Fixation

**Figure 1:**
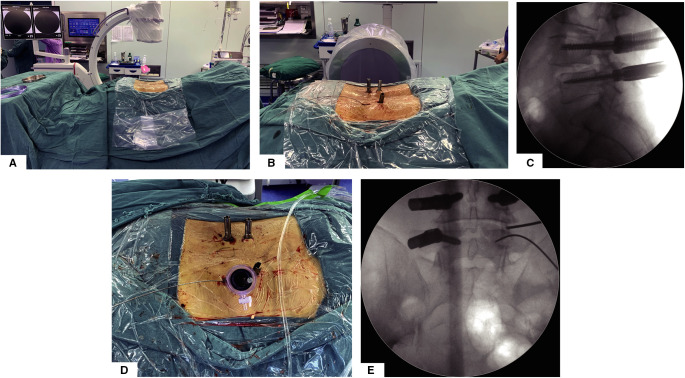
Surgery position, percutaneous pedicle screw fixation, and establishment of endoscopic working channel. (**A**) Prone surgery position and C-arm position. (**B**) General view of surgical incision and percutaneous pedicle screw implantation. (**C**) Lateral view of percutaneous pedicle screw fixation. (**D**) General view after establishing working channel. (**E**) AP view after establishing working channel.

All patients were placed in the prone position on a radiolucent table and the C-arm should be placed on the contralateral side of FE-PTLIF access (Figure 1A).

All operations were performed under general anesthesia and neuromonitoring.

Unlike the previously reported full-endoscopic intervertebral fusion surgery technique, our approach is more like microscopic TLIF; by this posterolateral transarticular approach, we do not need extra incisions for full-endoscopic decompression and fusion. Taking the right side of the L4/5 segment as an example, after completing the remaining three percutaneous pedicle screws, the guide wire for the L5 percutaneous pedicle screw will be retained (Figures 1B,C). Taking the implanted L5 pedicle screw incision as the incision, along the upper edge of the guide wire, we place the pencil tip on the superior articular process of L5 and gradually expand to establish the working channel (Figures 1D,E).

### Endoscopic Partial Facetectomy as Bone Graft and Decompression

**Figure 2:**
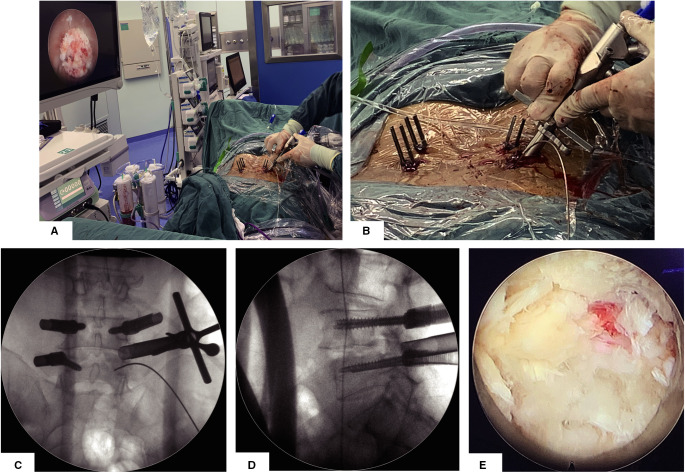
Articular osteotomy is performed by using this visualized trephine. (**A**) General view and endoscopic visual field after establishing the working channel for the visual trephine. (**B**) Detailed view when establishing the working channel for the visual trephine. (**C**,**D**) AP and lateral view after establishing the working channel for the visual trephine. (**E**) Endo-scopic view after articular osteotomy.

After establishing the working channel through the steps described above, the position will be confirmed by the anterior–posterior (AP) and lateral view of the x-ray. The surgeon can see the surface of the facet joint after clearing soft tissue via endoscopic visualization. Once the facet joint is identified according to the anatomy of the articular surface, osteotomy on the superior half of the superior articular process is performed by using this visualized trephine (Figures 2A,B). We will confirm the position of the visual trephine through AP-lateral fluoroscopy and the endoscopic anatomical structure (Figures 2C,D) and then perform sufficient articular process through the visual trephine to explore the nerve roots and prepare sufficient space for the working tube (Supplementary Video 1); partial facetectomy is efficient, convenient, and safe for whole osteotomy to be visible, and the bony fragments can be used as a bone graft for fusion. After removing part of the facet joints, the surgeon will remove part of the ligamentum flavum, intervertebral disc, and posterior longitudinal ligament to complete the exposure and decompression of the traversing nerve root and dural sac (Figure 2E). The osteotomy of the articular process is a necessary part of the full decompression of the nerve root and dural sac, which can provide bone grafting material for intervertebral fusion and provide enough space for cage insertion. If the patient has bilateral neurological symptoms, we need to take a contralateral inferior percutaneous pedicle screw incision for adequate percutaneous endoscopic decompression via the same approach as a supplementary surgery. Similar to conventional endoscopic decompression surgery, we usually take nerve root relaxation, visible nerve root pulse with the dural sac, and no obvious compression as the decompression standard.

### Endplate Preparation, Bone Graft, and Cage Insertion

**Figure 3:**
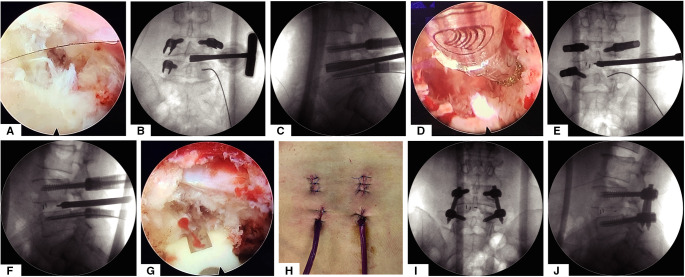
The whole process of decompression and intervertebral disc treatment and implantation of intervertebral fusion cage under the visual channel. (**A**) Endoscope visual field assisted by visual working channel after articular process osteotomy and decompression of the ligamentum flavum. (**B**,**C**) The AP and lateral view of x-ray for discectomy by various paddle distractor. (**D**) Endoscopic view of endplate preparation by using turnable burrs. (**E**,**F**) Implant an intervertebral fusion cage under the guidance of AP and lateral view of x-ray. (**G**) Endoscopic view after implanting the intervertebral fusion cage. (**H**) General view of the postoperative incision. (**I**,**J**) AP and lateral view after surgery.

The replaced custom-made endoscopic working tube is settled to block dura, the exiting and traversing nerve root out (Figure 3A and Supplementary Video 2). The custom-made working tube is a flexible, transparent plastic of several sizes, as shown in the video (Supplementary Video 2); the surgeon gently pushed the nerve root out of the operating space by the custom-made working tube, which can be stuck in the intervertebral foramen of the channel, and the operating space does not need to be very round or too large for the flexibility of the tube. After placing the customized working channel in place, the conventional paddle distractor, ring, and endplate curettes can be used to remove the disc efficiently and safely, the AP and lateral view of the x-ray for various paddle distractors will decide the size of the cage and range of endplate preparation (Figures 3B,C). Incomplete endplate preparation may result in fusion failure; endoscopic burr can be used as a supplementary tool to ensure the adequacy of endplate preparation under endoscopic visualization (Figure 3D); allograft and the autogenous bone retained from facet joint osteotomy will be placed into the anterior disc space through a regular funnel-shaped bone graft device. The conventional TLIF peek cage (kidney-shaped design) will be inserted into the intervertebral space under AP-lateral fluoroscopy (Figures 3E,F). The surgeon can reconfirm the position of the cage and decompression of the nerve root under endoscopic visualization (Figure 3G). The last percutaneous pedicle screw will be inserted by the guide wire and the rod will be inserted from the upper incisions for percutaneous screws, a small drainage catheter was finally inserted to prevent postoperative epidural hematoma(Figures 3H–J). This custom-made endoscopic working tube is a very useful tool, which can provide a safe space for the use of conventional tools, without the need to use additional special instruments or an intervertebral cage, and at the same time, the operation is more convenient and effective.

#### Application of the Custom-Made Working Channel

**Figure 4:**
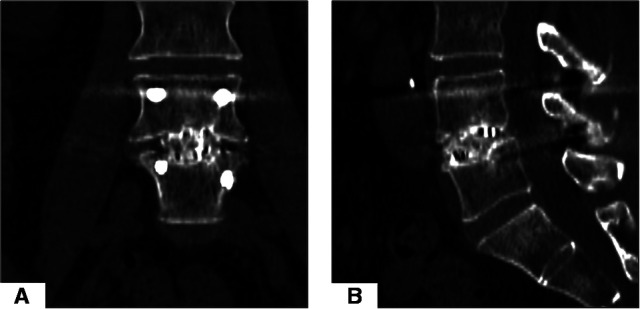
CT scan for three months after surgery. (**A**) Coronary scanning. (**B**) Sagittal scanning.

As mentioned before, this working channel was developed for FELIF. When using the traditional working channel, we often worry about whether the nerves are compressed outside the field of vision. At first, because the size of the 10 ml syringe was just right, its inner diameter was about 16 mm, and it could just cut the front end of the 10 ml of syringe into a duckbill opening through the intervertebral fusion cage that does not exceed 13 mm in height, and then, we use this homemade syringe as a working channel (Figure 4A). However, due to the limitation of the length and a single diameter model, we have designed a working channel of different diameters and lengths, and we have declared a patent based on this. As shown in Figure 4B, the schematic diagram of the section and each face of the working channel showed a similar structure and material to a homemade syringe but with more detailed tick marks. Besides that, we also designed a matching pencil tip in the patent. The schematic diagram in Figure 4C shows the cross section of the matching pencil tip, and the inner core can be placed with a 2 mm K-wire. Some differences between the custom-made and traditional working channels are particularly shown in Table 1.

**Table 1:** Differences between the custom-made and traditional working channels.

	Custom-made	Traditional
Material	Plastic	Metal
Reusability	Disposable	Reusable
Visibility	Visible	Invisible
Flexibility	Kind of flexible	Rigid
Scale mark	Yes	No

#### Analysis of Clinical Results

We recruited a total of 39 patients who only needed single-segment fusion surgery, all patients were followed up for more than nine months. Diagnosis, operative time, estimated blood loss, general data, and complications were evaluated. The visual analog scale (VAS) and Oswestry Disability Index (ODI) were evaluated during the preoperative and postoperative periods. All enrolled patients signed relevant surgical consent and informed consent. Statistical analyses were performed using SPSS version19.0 statistical software (SPSS, Inc., Chicago, IL). Quantitative data are expressed as *x̅* ± *s*. A *t*-test was used to compare differences between two groups. *P* < 0.05 was considered statistically significant.

## Results

A total of 39 patients (17 men and 22 women; mean age [*x̅* ± *s*] 55.2 ± 12.2 years) have been enrolled in this study since March 2019. The mean follow-up period was 11.5 ± 8.1 months. A total of 39 vertebral levels in 39 patients were treated using fully endoscopic posterolateral transarticular lumbar interbody fusion; 26 patients had degenerative spondylolisthesis, 6 patients had central stenosis with segmental instability, 4 patients had central stenosis with concomitant foraminal stenosis, and 3 patients had isthmic spondylolisthesis. The operative levels focused on L4/5 to L5/S1: L4/5 in 21 patients and L5/S1 in 18 patients (Table 2).

**Table 2:** Patient characteristics.

Characteristic	Value
Mean age (years)	55.2 ± 12.2
Sex	
M	17
F	22
Mean follow-up period (months)	11.5 ± 8.1
Level treated	
L4/5	21
L5/S1	18
Diagnosis	
Degenerative spondylolisthesis	26
Isthmic spondylolisthesis	3
Central stenosis w/ segmental instability	6
Central stenosis w/ concomitant foraminal stenosis	4
Mean estimated blood loss (ml)	54.4 ± 20.3
Mean operative time (mins)	130.5 ± 23.8
Postop complications	
Numbness	6

VAS and ODI scores improved significantly after surgery. The VAS scores decreased from 7.26 ± 1.23 preoperatively to 1.44 ± 1.04 at the last follow-up visit (*p* < 0.05), and the ODI scores decreased from 41.38 ± 5.36 to 7.28 ± 2.15 (*p* < 0.05). No patients experienced deterioration of neurological function after surgery. The mean total blood loss (including drainage blood) was 54.4 ± 20.3 ml. The mean operative time was 130.5 ± 23.8 min.

Six patients experienced numbness in the corresponding segmental distribution area after the operation, but all recovered spontaneously within 3 months. No patients had serious postoperative complications and none of these patients required revision surgery during the perioperative or follow-up period.

**Figure 5:**
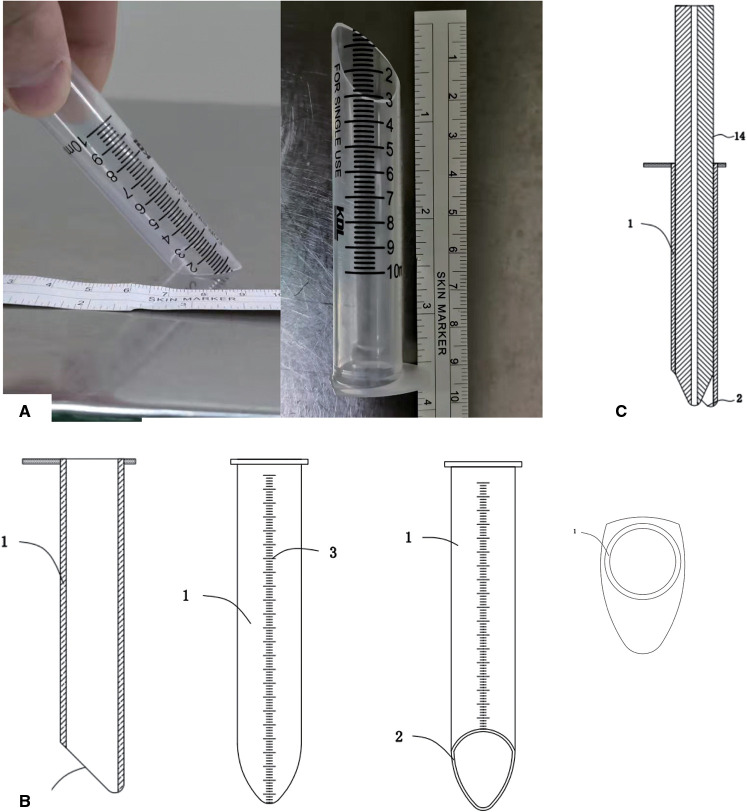
Custom-made working tube. (**A**) Homemade working channel with 10 ml syringe. (**B**) Schematic diagram of the cross-section and each side view of the custom-made working channel. (**C**) Schematic diagram of the cross-sectional view of the pencil tip and custom-made working channel.

A total of 39 enrolled patients were observed intervertebral fusion at the last follow-up. Our criteria for judging intervertebral fusion include no obvious active low back pain and a CT scan showing the bone connection in the intervertebral space. Figure 5 shows the imaging manifestations of typical cases during postoperative CT follow-up.

## Discussion

Due to substantial technological advancements in minimally invasive spinal surgery, endoscopic TLIF has become accessible in clinical practice. Compared with traditional open spinal fusion surgery, endoscopic TLIF does less damage to soft and bone tissues, has less blood loss, has faster recovery, has clearer vision under the endoscope, has more adequate treatment of nerve decompression, and has endplate preparation to increase the chance of intervertebral fusion and make the effect more accurate (10). In this study, FE-PTLIF adopts the posterolateral transforaminal approach, which can obtain an appropriate amount of autogenous bone during surgery and get a better decompression for the nerve root and dura. We can use conventional operation instruments and fusion devices to make operation more convenient and safe without increasing the cost to the patient by using this custom-made transparent plastic working tube.

Like the reports of endoscopic TLIF surgery in recent years, all the 39 patients in this study achieved very satisfactory clinical results and intervertebral fusion, and there were no related complications. The most commonly reported complications of endoscopic TLIF surgery include dural tear, infection, and epidural hematoma (11, 12); although there is a lack of prospective randomized controlled studies, the currently available case series and comparative studies seem to support a lower overall complication rate of endoscopic TLIF surgery compared to their MIS or traditional spinal surgery (13). Furthermore, endoscopic TLIF can be distinguished into three surgical techniques based on the type of the endoscope used (percutaneous endoscopic TLIF with a working channel, biportal endoscopic TLIF, microendoscopic TLIF, and Full-Endoscopic Oblique Lateral Lumbar Interbody Fusion) (14). Almost all these studies mentioned the problem of the steep and potentially dangerous learning curve (11, 15); the possible reason includes (1) the anatomy of the intervertebral foramina under the endoscope is unfamiliar (16), and the risk of exiting nerve root injury is high, especially during the placement of the cage, so there are some reports in the literature about expandable mesh interbody fusion cage (4, 17); its main advantages appear to be decreased anatomical disruption during delivery and deployment. The problem is that this will increase the financial burden on the patients, and a larger number of patients and further long-term follow-up are warranted (18). (2) Surgical operation time is too long (19), especially in early cases, which may be safer for osteotomy of the articular process and endplate preparation by using burrs under the endoscopic visualization, but with a lower efficiency; to overcome the above-mentioned problems as much as possible, we adopted this posterolateral transforaminal approach, which is similar as the traditional open surgical approach, surgeons may be more familiar with the anatomy to get a better posterior decompression than regular endoscopic TLIF, and we can obtain autogenous bone for bone grafting during facetectomy to expect a higher fusion rate, the application of this novel transparent plastic channel can be equipped with conventional instruments, making the surgical operation more efficient and safe, and will not increase the burden of the patient compared with endoscopic lumbar interbody fusion by using expandable cage.

Kenji et al. mentioned the problem of excessive radiation exposure, which may increase the risk of health problems for the surgical team and the patients (20, 21), In our research, skilled surgeons can stay behind the lead screen when radiation exposure is needed, so the surgical team does not require radiation exposure in the whole process. However, the patient’s radiation exposure is higher than that of traditional open TLIF surgery (20, 21).

Although our new surgical approach and instruments may make the learning curve smoother, there are still some limitations compared with traditional open TLIF surgery, which includes more radiation exposure to patients during surgery and there are still many spinal diseases that cannot be resolved by endoscopic surgery. Also, because of the use of percutaneous screws and endoscopy, it will still increase the burden on some patients. Clinical study of additional pedicle screw fixation. In the next study, we may consider the clinical study of pure intervertebral fusion without additional pedicle screw fixation.

## Conclusion

FE-PTLIF surgery has the advantages of less trauma and faster recovery because of its clear vision, enough decompression, adequate endplate preparation, and autologous bone graft materials can be obtained during the operation. Our preliminary clinical results also showed that this surgical method has a good fusion rate and clinical efficacy. In general, FE-PTLIF is a safe and effective interbody fusion option for most lumbar degenerative diseases, which can be equipped with conventional instruments by using a transparent plastic working tube.

## Data Availability

The raw data supporting the conclusions of this article/Supplementary Material will be made available by the authors, without undue reservation.

## Supplementary Material

**Supplementary Video S1** | Process of arthroplasty and exposure of nerve roots with a visual trephine by posterolateral transarticular approach.

**Supplementary Video S2** | Application of self-made visual working channel under endoscope.
